# Reducing Ochratoxin A Content in Grape Pomace by Different Methods

**DOI:** 10.3390/toxins12070424

**Published:** 2020-06-27

**Authors:** Jianmei Yu, Ivy N. Smith, Nona Mikiashvili

**Affiliations:** Department of Family and Consumer Sciences, North Carolina Agricultural and Technical State University, Greensboro, NC 27411, USA; ivyn.smith@gmail.com (I.N.S.); nmikiash@ncat.edu (N.M.)

**Keywords:** ochratoxin A, OTA reduction, thermal pressure treatment, acid treatment, enzymatic treatment

## Abstract

Grape pomace (GP) is the residue of grapes after wine making and is a valuable source of dietary polyphenol and fiber for health promotion. However, studies found the presence of ochratoxin A (OTA) in GP at very high concentrations, which raises a safety issue in the value-added utilization of GP. This study evaluated the effects of thermal pressure, baking, acid and enzymatic treatments on OTA content in GP. Thermal pressure treatment was conducted with wet GP at 121 °C for 10–30 min in an autoclave; acid treatments were conducted with hydrochloric acid, acetic acid, citric acid, and lactic acid, respectively, at 50 °C for 24 h. Baking was conducted using a cookie model. For enzymatic treatment, purified OTA solution was treated with carboxypeptidase A, alcalase, flavourzyme, pepsin, and lipase, respectively, and the effective enzymes were selected to treat GP. Results show that autoclaving for 10–30 min reduced 19–80% of OTA, varying with treatment time and GP variety. The effectiveness of acid treatment was similar to that of autoclaving and varied with acid type and GP variety. Baking increased the detectable OTA. Among all tested enzymes, carboxypeptidase A was the most effective in reducing OTA, followed by lipase and flavourzyme, but their effects were significantly lower in GP samples.

## 1. Introduction

Grapes are subject to mold contamination during cultivation, harvest, transport and/or storage. Molded grapes present a safety issue to products derived from grapes because of the presence of mycotoxins. The major mycotoxin in molded grapes is ochratoxin A (OTA) [1,2]. The most relevant OTA-producing species are *Penicillium verrucosum (P. verrucosum)*, *Aspergillus ochraceus (A. ochraceus)*, *A. niger* and *A. carbonarius* due to their prevalence in foodstuffs (cereals, grapes, coffee, etc.) [3]. Grape pomace is the residue of grapes after wine making and is a valuable source of phenolic antioxidants, dietary fiber and polyunsaturated lipids. Some of our studies show that GP has great potential to serve as an ingredient in food products such as bread, extruded breakfast and cookies at concentrations up to 5% (dry base) [4,5,6]. There is also increasing interest in using GP as a feed ingredient [7,8,9]. However, previous studies also found the presence of OTA-producing fungi (including *Aspergilus niger*, *A. carbonarius*, and *A. fumigatus*) and a high level of OTA in both wet and dry GP, which makes GP unsafe for human and animal consumption [10,11].

Dietary exposure to OTA represents a serious health issue and has been associated with several human and animal diseases, including poultry ochratoxicosis, porcine nephropathy, human endemic nephropathies and urinary tract tumors in humans [12]. Livestock consuming OTA-contaminated feed showed pale and grossly enlarged kidney, fatty liver in poultry, altered performance including decreased feed consumption, reduced weight gain, and decreased egg production; the intake of feeds contaminated by OTA was the probable cause of a disease named Denmark nephropathy in pigs. The sensitivities of livestock animals to OTA are in the order of pigs and dogs > poultry > calves > mature cattle [13,14]. In humans, various studies have linked OTA exposure with the human diseases Balkan endemic nephropathy (BEN) and chronic interstitial nephropathy (CIN), as well as other renal diseases [15]. New data available since the last risk assessment conducted by the European Food Safety Authority (EFSA) in 2006 suggest that OTA can be genotoxic by directly damaging DNA, and experts have also confirmed that OTA can be carcinogenic to the kidney [16].

Many countries have set limits for OTA, and concentrations need to be reduced to as low as technologically possible in food and feed. For example, in the European Union, a general maximum OTA limit is 5 µg/kg in cereals, 3 µg/kg in processed cereal products, and 10 µg/kg in dry vine fruits [17]. China’s OTA standard for cereal grains and legumes is 5 µg/kg [18]. The proposed Canadian OTA regulatory guide is the same as that established in Europe [19]. Likewise, Israel has applied a 50 µg/kg OTA standard to all cereals and pulses. Switzerland’s OTA standard is 2 µg/kg for all cereal products. Currently, the United States Food and Drug Administration (FDA) has not set regulatory guidelines for OTA in food or feed.

**Figure 1:**
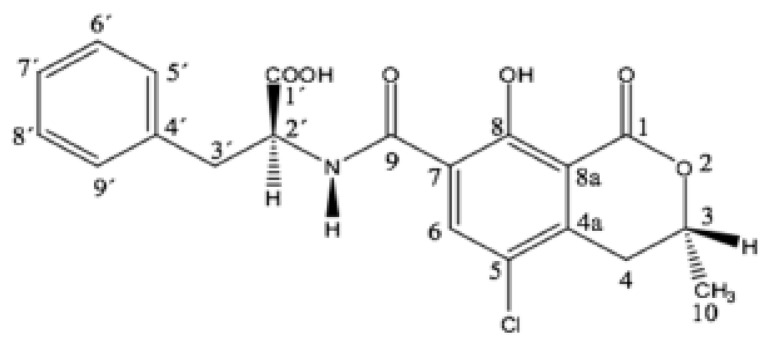
The chemical structure of ochratoxin A [21].

Structurally, OTA consists of a para-chlorophenolic group containing a dihydroisocoumarin moiety that is amide-linked to L-phenylalanine. Its chemical name is L-phenylalanine-N-[(5-chloro-3, 4-dihydro-8-hydroxy-3-methyl-1-oxo-1H-2-benzopyrane-7-yl) carbonyl]-(R)-isocoumarin, and its chemical structure is shown below (Figure 1) [20,21].

Although the most important strategy to control OTA level in food and feed is to prevent fungal growth and OTA production, detoxification becomes necessary if the food and feed materials are contaminated, in order to protect human and animal health, reduce food/feed waste, and even for safe disposal. For cereal grains and legumes, each physical processing step, such as sorting, sieving, floatation, washing, dehulling, milling, and heat treatment (such as cooking and roasting) can remove a certain amount of OTA [22,23]. The reported thermal transformation/degradation products of OTA are 2R’-OTA (called 14-(R)-ochratoxin A in the past), 14-decarboxy-ochratoxin A (DC-OTA) and ochratoxin alpha amide [24]. It was reported that gamma irradiation from 2 to 5 kGy effectively prevented the production of OTA or destroyed it when already produced, and carboxypeptidase at 5 units/50 mL in a liquid medium is very efficient for cleaving the OTA already produced [25]. Researchers have also discovered a good many microorganisms that could degrade and/or adsorb OTA, including actinobacteria, bacteria, filamentous fungi, and yeast; the degradation of OTA to non-toxic or less toxic OTα via the hydrolysis of the amide bond is the most important OTA biodegradation mechanism [26]. However, detoxification by microorganisms will cause unavoidable biochemical changes in food and feed stuff due to fermentation.

As a byproduct of grape processing, GP would theoretically have a higher mycotoxin level than the processed products, as in the case of brans of cereal grains [27,28]. Although our previous study demonstrated that vacuum drying not only inactivated molds, but also significantly reduced OTA content in GP [11], research on how to reduce OTA in GP is limited. Therefore, it is important to develop effective methods to destroy molds and transform OTA into less toxic or non-toxic compounds before the GP is added into food formulas to ensure food safety. This study investigated the effectiveness of some common food processing methods, including thermal pressure treatment, acid treatment, baking under slight alkaline conditions and enzymatic treatment on the OTA content of GP.

## 2. Results and Discussion

### 2.1. Effects of Thermal Pressure Treatment on the OTA Contents of Grape Pomace

**Figure 2:**
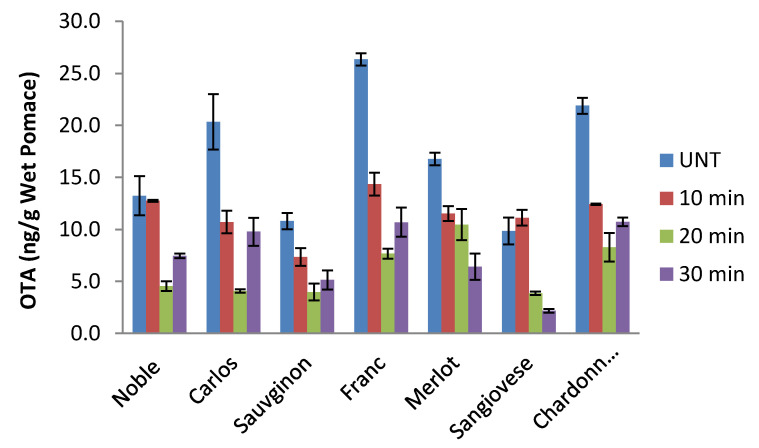
Ochratoxin A (OTA) content of wet grape pomaces (GPs) from different grape cultivars and effects of autoclaving time on OTA contents in wet grape pomace samples (UNT—untreated).

OTA was detected in all the tested grape pomace samples. The levels of OTA were 13–28 μg/kg in the wet pomaces, corresponding to 26–100 μg/kg in dry pomace. This is higher than the maximal allowance in raisin products (10 μg/kg) in Europe [17]. Thermal pressure treatment, which was conducted in an autoclave at 15 psi and 121 °C, significantly reduced total OTA content in grape pomace samples and the degree of OTA reduction was dependent on autoclaving time; the highest OTA reduction for most of the GP samples was achieved at 20 min (Figure 2). Among seven GP samples, the OTA contents of six samples were lower than 10µg/kg GP, which is lower than the maximum allowance in raisin. The efficiency of autoclave on OTA reduction varied with the type of GP (Table 1). The OTA reductions at treatment times of 10, 20 and 30 min were 19.21–67.87%, 37.64–79.88% and 43.77–78.01%, respectively.

The presence of amide bonds in the chemical structure of OTA makes it possible to breakdown this bond to form L-phenylalanine and the less-toxic ochratoxin α by different methods, although OTA has been reported to be stable to heat and acidic conditions [29]. It is known that the thermal degradation products of OTA are 14-(R)-ochratoxin A (now called 2′R-OTA), 14-decarboxy-ochratoxin A (DC-OTA) and ochratoxin alpha amide (OTα-amide), all of which have reduced toxicity [30,31]. Sufficiently high temperature and heating time are necessary to cause OTA degradation. A study by Park et al. found that washing polished rice with water had little effect on OTA levels, but cooking significantly reduced OTA concentration in the rice, and the OTA levels remaining in ordinarily cooked rice and pressure-cooked rice were 69–75% and 59–60%, respectively [32]. A study by Boudra and colleagues found that dry heating of OTA-contaminated wheat at 100 °C for 40–160 min did not change OTA content, whereas, upon wet heating at the same temperature, over 50% of the OTA was destroyed after 120 min [33]. In our study, wet GP samples were heated in an autoclave to 121 °C and maintained for 10–30 min under high pressure (15 psi), and results are in good agreement with those reported in the literature [32,33]. In addition to reducing OTA, thermal pressure processing will also eliminate all microorganisms in the GP. Therefore, thermal pressure processing such as pressure cooking and pressure steaming could be an effective method to improve the safety of GP, and thus the safety of food/feed containing GP. However, it can be seen from Figure 2 and Table 1 that that OTA contents in some GP samples autoclaved for 30 min were higher than those autoclaved for 20 min. This might be caused by the interference of other compounds formed during heating on the determination of OTA. For instance, it was reported that isomerization of OTA occurred during coffee roasting and formed 2R’-OTA, which was detected by LC-MS/MS [24]. Considering the structural similarity between OTA and 2R’-OTA, they might not be differentiated by ELISA. Hence, the OTA content determined by ELISA in the thermal-pressure-treated GP sample could be the sum of OTA and 2R’-OTA, and longer treatment time could contribute to higher 2R’-OTA content. Thus, the higher OTA in the GP samples autoclaved for 30 min might be overestimated.

### 2.2. Effects of Thermal Pressure Treatment on Polyphenol Contents of Grape Pomace

**Figure 3:**
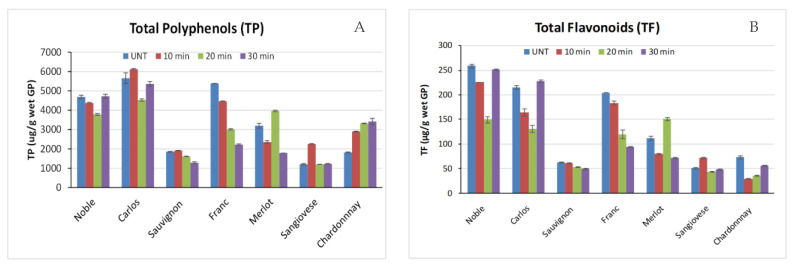
Effects of thermal pressure processing (autoclaving) time on total polyphenol (**A**) and flavonoid (**B**) contents of grape pomace.

The effect of high-pressure thermal treatment on polyphenols of grape pomace was tested. Overall, autoclaving resulted in decreases in total polyphenol (TP) and total flavonoids (TF) in some GP samples (Muscadine Noble and Carlos, Cabernet Sauvignon and Franc, as well as Merlot), did not change TP and TF in Sangiovese GP, and increased TP in Chardonnay GP (Figure 3). However, the loss of polyphenols due to autoclaving is acceptable given their high contents in GP. This may be attributed to the relative stability of polyphenols at acidic pH [34,35,36] because the pH of GP slurry is about 4.5. In addition, autoclaving was conducted in a well-sealed container, and a large quantity of vapor was generated from water within the sample as the temperature increased, which exiled the oxygen trapped in the sample and reduced the oxidation degradation of polyphenols, the main degradation pathway.

### 2.3. Effects of Acid Treatments on OTA Content in GP

In this study, all GP samples were treated at same pH and temperature for the same period of time (24 h). Data in Table 2 show the effectiveness of different acids on OTA reduction in GP. Overall, organic acids including acetic acid (AA), citric acid (CA) and lactic acid (LA) were more effective in reducing OTA in all grape pomaces than hydrochloric acid (HCl), which was only effective in reducing OTA in Cabernet Sauvignon. Among the three organic acids, it is hard to tell which one is more effective. Treatment of GP under highly acidic conditions for sufficient time will also completely inactivate bacteria and fungi in the GP and prevent further production of any toxin. Table 2 also shows that the OTA-reducing efficiency of same acid varied with GP variety, which might be related to the interference of the polyphenol composition of the GP with OTA quantification, although no such study has been reported.

An early study found that, when heated under moist and watery conditions, a small change in OTA molecule was found, but cytotoxicity was not reduced. Under acidic conditions (0.1 HCl), the decomposition of ochratoxin A was detected by TLC; however, a change in cytotoxicity was not observed. On the other hand, heating with NaOH (0.1 N) resulted in the decomposition and detoxification of ochratoxin A [37]. A recent study conducted in buffer solutions of different pHs (pH 4, 7 and 10) found that the rate and the extent of OTA reduction were dependent on pH, processing time, and temperature: an OTA reduction greater than 90% was achieved at 200 °C for all treatments except pH 4; about 50% of the OTA was lost after treatment at pH 10 and 100 °C for 60 min, while a significant OTA reduction was not observed after 60 min under neutral and acidic conditions at 100 °C [38]. However, the reduction of OTA under alkaline conditions might be due to the reversible conversion of OTA to the open lactone of ochratoxin A (OP-OTA), which was found to be more toxic than OTA in rats and mice [13,39]. The autoclaving treatment used in the present study was conducted at the pH of GP (near pH 4.0), and the highest OTA reduction achieved was 67%. More study is needed to test if a longer acidic treatment time at room temperature or a shorter treatment time at a higher temperature can achieve a higher OTA reduction in GP.

### 2.4. Effects of Baking on OTA Contents

**Figure 4:**
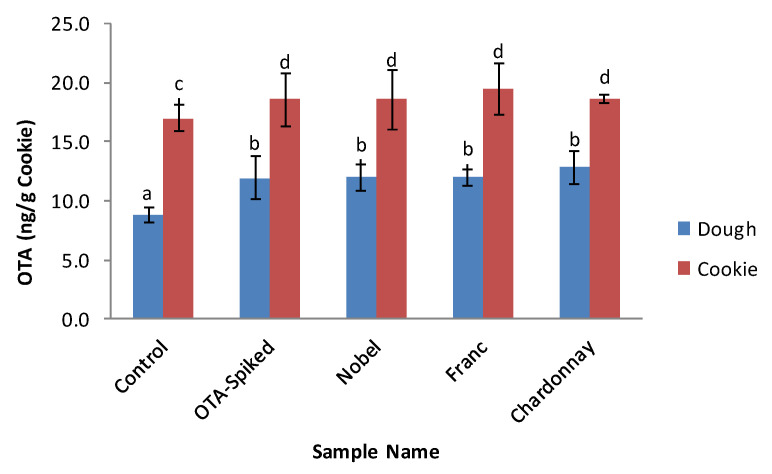
OTA contents of dough and cookies with and without GP-containing cookies. (All GP-containing samples were spiked with OTA. Noble—Muscadine Noble, Franc—Cabernet Franc). (Different letters on data bars indicate significantly different values at *p* < 0.05).

Because OTA is commonly present in cereal grains and the products made from cereal grains, the OTA contents of cookie dough and cookies without added GP or OTA spiking solution were determined and used as controls. The OTA content of the all-purpose flour used for cookie making was detected to be 9.44 ng/g flour. Figure 4 shows that the dough without OTA spiking or GP (control 1) contained 8.82 ng/g of OTA (dry base). Regardless of GP addition, all cookies showed higher OTA contents than their corresponding dough, and there were no significant differences in OTA content among dough and cookies spiked with OTA. Compared with dough, baking resulted in a 45.4–69.8% increase in OTA. The data indicate that the wheat flour used for cookie making in this study was contaminated with OTA, the contribution of GP addition (5%) to the OTA content of dough and cookies was negligible, and cookie baking increased the detectable OTA content in the cookies.

The results of this study disagree with most of the data reported in the literature, but agree with the results of Vidal and colleagues who reported a 40% increase in OTA from dough to bread [40]. It is well known that ochratoxin is stable during bread baking, but baking of biscuits was reported to result in about two thirds of the toxin being destroyed or immobilized [22]. The pH of bread dough is usually in the range of 4.5–6.0, while the pH of biscuit or cookie dough is 7.0–7.2. OTA should be stable at both pH ranges as demonstrated by [38]. Therefore, it is no surprise that the cookie baking process did not reduce OTA in this study. The increase in OTA after baking may be explained by the increase in OTA extractability or the formation of other compounds which could bind to the antibody in the ELISA kit, thus resulting in overestimation of OTA. Analyzing OTA content by different methods, such as HPLC, may produce different results.

### 2.5. Effects of Enzymatic Treatment on OTA Content in Grape Pomace

**Figure 5:**
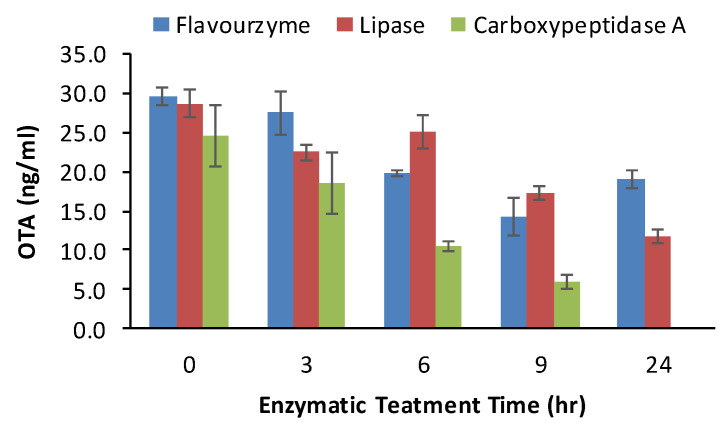
Effects of enzymatic treatment on OTA content in buffer solution.

**Figure 6:**
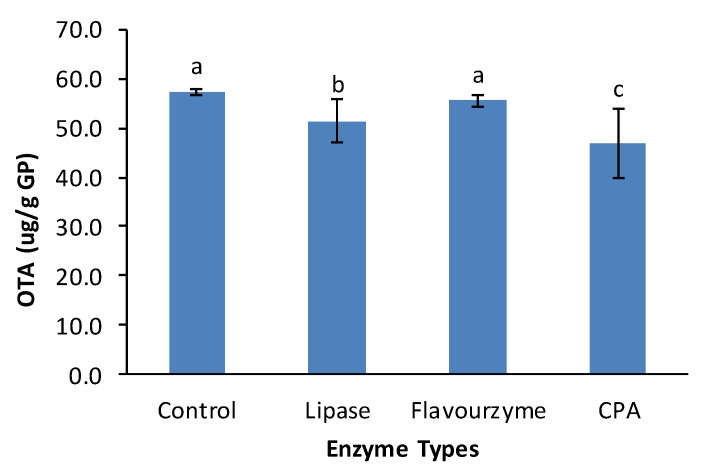
Effects of enzymatic treatment on OTA content in grape pomace (37 °C for 24 h). (Different letters on data bars indicate significantly different values at *p* < 0.05).

In this study, the potential of carboxypeptidase A (CPA), alcalase, flavourzyme (protease from Aspegillus niger), lipase, and pepsin to reduce OTA content was first screened using pure OTA solution. Among all tested enzymes, only CPA, flavourzyme and lipase significantly reduced OTA concentration in the buffer solutions, and the reductions of pure OTA due to treatment with flavourzyme, lipase and CPA at 37 °C for 24 h were 36, 60 and 100%, respectively (Figure 5). Therefore, these three enzymes were used to treat GP samples containing known amounts of OTA. Figure 6 shows that lipase and carboxypeptidase A treatment significantly reduced OTA contents in the GP samples (*p* < 0.05), but the reductions were only 10.22% and 18.33%, respectively, whereas flavourzyme treatment did reduce OTA.

Some commercial enzymes, including lipases and proteases from *Aspegillus niger*, were reported to effectively hydrolyze OTA into less toxic OTα and β-phenylalanine [32,41]. However, the results of this study showed that CPA was highly effective and can completely hydrolyze OTA in pH 5.4 buffer, but the efficacy of this enzyme was limited in reducing OTA in grape pomace. The effects of lipase and protease from *A. niger* on OTA in GP were also limited, although statistically significant. This might be caused by the interference of GP polyphenols, because grape pomace/seed polyphenols also function as inhibitors that inhibit the activities of different hydrolytic enzymes, such as protease, lipase and carbohydrase [42]. Longer treatment time may increase OTA reduction but it may also increase OTA content if the GP is not sterilized before enzyme treatment because of the presence of viable OTA-producing molds in GP [10].

## 3. Conclusion and Implication

This study demonstrated that thermal pressure processing, such as autoclave and pressure cooking, could effectively destroy OTA in grape pomace without causing too much damage to polyphenols, but the time of treatment has to be controlled to avoid excess destruction of polyphenols. Treatment using organic acids, such as acetic and citric acid, at concentrations of 0.01 M (pH 2.0) also reduced OTA in GP significantly. Similar to breadmaking, cookie baking could not reduce OTA. Although hydrolytic enzymes such as carboxypeptidase, lipase and protease from *Aspegillus niger* showed great potential to reduce OTA in the buffer solutions, their efficacies in OTA reduction in GP were very limited, even when the treatment time was 24 h. Therefore, enzyme treatment alone may not be an effective approach for reducing OTA in GP; the combination of thermal pressure treatment and acid/enzyme treatment may reduce OTA further, and may be worth further study. Because most of the degradation products of OTA are reported as being less toxic, it is reasonable to assume that GP treated by thermal processing, acid and enzymes should be safer than untreated GP. However, this assumption needs to be tested by in-vitro and in-vivo toxicity studies using cell cultures and animal model. The limitation of this study is that OTA was quantified by ELISA, which displays high variation and cannot provide information about the degradation products of OTA. More consistent and accurate methods, such as high-performance liquid chromatography (HPLC) or LC-MS/MS, are needed in future studies related to the degradation or transformation of OTA.

## 4. Materials and Methods

### 4.1. Materials

The wet grape pomace samples from seven grape cultivars, including Muscadine Carlos, organic Muscadine Noble, organic Cabernet Franc, Cabernet Sauvignon, Merlot, Sangiovese and Chardonnay were obtained from two North Carolina wineries. They were collected right after press, packed in gallon-size plastic bags separately and stored at −20 °C until use. Purified ochratoxin A (lyophilized powder) from *Aspergillus ochraceus*, carboxypeptidase A from bovine pancreas, lipase and protease from *A. niger*, alcalase from *Bacillus licheniformis*, and pepsin from porcine gastric mucosa were purchased from Sigma-Aldrich (St. Louis, MO, USA). Acetic acid, citric acid, lactic acid and hydrochloric acid were purchased from Fisher Scientific (Suwanee, GA, USA).

### 4.2. Treatments of Grape Pomace and OTA Extraction

#### 4.2.1. Thermal Pressure Treatment

The GP samples were thawed overnight and 100 g of each was weighed into a set of three containers, capped, then autoclaved for 10, 20 and 30 min at 15 psi and 121 °C in a laboratory autoclave. The untreated pomace samples were used as controls. After cooling, 100 mL of DI water was added to each container, and the GP samples were homogenized. OTA was extracted using undiluted methanol. Briefly, 10 g of GP slurry was mixed with 20 mL of methanol in a 50-mL Erlenmeyer flask for 30 min on a magnetic stir, and centrifuged at 3000× *g* for 20 min. The supernatant was collected for OTA determination. The extraction was conducted in triplicate for each sample.

#### 4.2.2. Acid Treatment

The reason to select acid treatment is that most of polyphenols (anthocyanins, catechins and flavonoids) in GP are stable under acidic conditions even at high temperatures [34,35,36]. The GP samples were treated with acetic acid (AA), citric acid (CA), lactic acid (LA) and hydrochloric acid (HCl) at pH 2.0, respectively, for 24 h at 37 °C. Briefly, 50 g of wet GP was suspended in 0.01 M of acid solution in a glass container at a GP-to-acid ratio of 1:1 (w/v) and adjusted to pH 2 using 2 M HCl and 2 M NaOH, then incubated at 50 °C for 3, 6, 9, 18 and 24 h. GP samples without added acid were used as the control. After homogenization, OTA was extracted using 100% methanol as described in Section 4.2.1.

#### 4.2.3. Baking

According to our previous studies, cookies are one of the food products suitable for GP application [4,5,6]; thus, cookie making was used as the baking model in this study. The vacuum-dried GP was ground and sieved, and the portion passed through a 40-mesh screen was used for baking. The OTA content of GP powder and flour were determined before cookie making. Cookie dough was formulated with all-purpose flour, sugar, butter, egg, baking soda, vanilla extract, and 5% GP with known OTA content. The dough was then spiked with 5 ppb of OTA and mixed thoroughly with a kitchen aid mixer; the dough was then divided into 6 equal balls, 3 were baked at 350 °F (178 °C) for 20 min in a lab oven, and 3 were used to determine total OTA before baking. The dough and cookie samples spiked with OTA but without GP were used as controls. For OTA extraction, the dough was homogenized with 80% methanol, while cookie was broken into small pieces, ground into powder, and then extracted with 80% methanol. The moisture of dough and cookie were determined by drying spread samples in a vacuum oven for 24 h at 80 °C.

#### 4.2.4. Enzymatic Treatment

Alcalase, flavourzyme, lipase, pepsin, and carboxypeptidase A were used for enzymatic treatment. For lipase, alcalase and flavourzyme, the treatments were conducted in pH 7.5 phosphate buffer (PB), the pepsin treatment was conducted in simulated gastric fluid at pH 1.5, papain treatment was conducted in PB at pH 6, and carboxypeptidase treatment was conducted in Tris buffer at pH 8.0. Purified OTA solution was diluted to 30 ng/mL in a set of test tubes with different buffers containing different enzymes. The enzyme concentration was 10 mg per 1µg OTA as described by Abrunhosa and colleagues [43]. The treatment was conducted at 37 °C in a water bath shaker for 0–24 h at the optimal pH of that enzyme, and samples were taken at 0, 3, 6, 9 and 24 h. After inactivation of the enzyme in a boiling water bath, the OTA concentration was determined by using an ELISA kit. The enzymes that resulted in obvious OTA reduction were selected to treat GP. Before enzyme treatment, the GP was sterilized by autoclaving to inactivate microorganisms, followed by homogenization to ensure the even distribution of spiked OTA and enzyme. The OTA content of the GP slurry was determined, and slurry was then spiked with 10 ng/g of OTA. After thorough mixing, the selected enzyme was added and GP slurry samples were incubated in the water bath shaker for 24 h at 37 °C followed by enzyme inactivation. OTA-spiked GP slurry without added enzyme but incubated under same conditions was used as a control. The OTA in treated GP samples was extracted in triplicate as described in Section 4.2.1.

### 4.3. OTA Determination

The OTA contents of extracts were determined in triplicate by a rapid immune assay using the AgraQuant^®^ Ochratoxin Assay Kit (RomerLabs, Newark, DE, USA) according to the manufacturer’s instructions. All OTA extracts were adjusted to pH 7.0 ± 0.10 using 2 M HCl or 2 M NaOH, centrifuged again to remove any particles, and quantitatively diluted with 80% methanol before ELISA assay. The OTA content was expressed as ng/kg sample according to sample weight, extract volume and dilution factor. The OTA contents of dough and cookies were based on dry sample weight.

### 4.4. Polyphenol Extraction and Analysis

The polyphenols in treated and control samples were extracted in the same way as for OTA extraction. Total polyphenol (TP) was determined by the Folin–Ciocalteu method [44] modified for microplate assay. Total flavonoids (TF) was determined by the aluminum chloride (AlCl_3_) colorimetric method [45].

### 4.5. Data Analysis

Data were analyzed by post-ANOVA Duncan test using SAS version 9.4 (SAS Institute, Cary, NC, USA). The percentages of OTA reduction under specific treatments were calculated for each sample.

## Figures and Tables

**Table 1:** Percent OTA reduction in grape pomace as affected by different autoclaving times.

Variety of Grape Pomace	OTA Reduction (%)		
	10 min	20 min	30 min
Muscadine Noble	21.64	65.77	43.77
Muscadine Carlos	62.93	79.98	52.03
Cabernet Sauvignon	67.60	63.29	52.57
Cabernet Franc	47.89	70.95	59.46
Merlot	47.49	37.64	61.87
Sangiovese	19.21	61.03	78.01
Chardonnay	55.71	62.23	50.97

**Table 2:** Reduction of OTA contents in acid-treated grape pomace samples.

Treatment	Cabernet Franc	Reduction (%)	Cabernet Sauvignon	Reduction (%)	Chardonnay	Reduction (%)
Control	25.11 ± 3.15	0.00	14.15 ± 0.79	0.00	21.68 ± 2.59	0.00
AA	10.09 ± 3.22	59.82	12.07 ± 2.57	14.68	8.36 ± 4.53	61.42
CA	10.84 ± 0.31	56.84	6.12 ± 5.44	56.78	17.29 ± 1.91	20.23
HCl	23.07 ± 0.12	8.12	5.27 ± 2.72	62.77	16.27 ± 0.73	24.95
LA	26.20 ± 0.42	−4.35	4.64 ± 0.54	67.23	11.62 ± 3.29	46.39

AA—acetic acid, CA—citric acid, HCl—hydrochloric acid, LA—lactic acid.
